# Adipocyte Ferroptosis in Obesity: Molecular Mechanisms and Nutraceutical Perspectives

**DOI:** 10.3390/ijms27188357

**Published:** 2026-09-19

**Authors:** Stefano Ruga, Elisa Matarese, Antea Maria Pia Mangano, Chiara Lamesta, Tiziana Dimatteo, Leonardo Miscio, Canio Martinelli, Marilena Lauriola, Renato Lombardi, Antonio Giordano, Giovanna Liguori

**Affiliations:** 1 Department of Health Sciences, Magna Graecia University of Catanzaro, 88100 Catanzaro, Italy; 2 Vita-Salute San Raffaele University, 20132 Milan, Italy; 3 Local Health Authority of Foggia (ASL Foggia), 71121 Foggia, Italygiovanna.liguori@aslfg.it (G.L.); 4 Sbarro Institute for Cancer Research and Molecular Medicine, Center for Biotechnology, Department of Biology, College of Science and Technology, Temple University, Philadelphia, PA 19122, USA; 5 Sbarro Health Research Organization ETS, 10060 Candiolo, Italy; 6 Department of Medical Biotechnology, University of Siena, 53100 Siena, Italy

**Keywords:** glutathione peroxidase 4, Nrf2-Keap1 pathway, regulated cell death, polyphenols, insulin resistance, crown-like structures, inflammation, metabolic disorders, macrophage polarization, antioxidant defense

## Abstract

The global obesity epidemic underscores the urgent need to dissect the molecular mechanisms driving the transition from benign adipose tissue expansion to pathological dysfunction, with adipocyte death representing a critical tipping point in this process. This narrative review synthesizes evidence from peer-reviewed literature concerning the emerging role of ferroptosis in adipose tissue dysfunction, examining the core molecular machinery of iron-dependent lipid peroxidation, the GPX4-glutathione defense axis, and the Nrf2-Keap1 cytoprotective pathway within the specific context of obese adipose tissue biology. The analysis reveals that the obese adipose microenvironment, characterized by pathological iron accumulation, enrichment of peroxidation-prone polyunsaturated fatty acid-containing phospholipids, and a chronically besieged antioxidant defense network, creates conditions uniquely favorable to ferroptotic execution. Furthermore, available evidence suggests that signals released from ferroptotic adipocytes are likely to promote macrophage polarization toward a pro-inflammatory phenotype, establishing a self-amplifying pathogenic loop that propagates local and systemic metabolic dysregulation. The review identifies multiple plant-derived nutraceuticals, including curcumin, resveratrol, quercetin, bergamot polyphenolic fraction, sulforaphane, oleuropein, and astaxanthin, as promising multi-targeted modulators capable of intercepting the ferroptotic cascade at the levels of iron catalysis, lipid radical propagation, and Nrf2-dependent antioxidant defense potentiation. These findings position adipocyte ferroptosis as a novel therapeutic target in obesity and support the rationale for mechanism-based nutraceutical interventions aimed at protecting the adipose organ from pathological cell death.

## 1. Introduction: Adipose Tissue Expansion, Hypoxia, and the Limits of Healthy Adipose Remodeling

The global prevalence of obesity has reached epidemic proportions, establishing it as one of the most pressing public health challenges of the 21st century. Obesity is a complex, multifactorial disease characterized by the excessive and dysfunctional accumulation of adipose tissue, which significantly elevates the risk of developing a wide spectrum of associated pathologies, including type 2 diabetes, cardiovascular disease, non-alcoholic fatty liver disease (NAFLD), and several types of cancer [1,2,3]. Despite considerable efforts to combat the obesogenic environment, the number of affected individuals continues to rise, underscoring the urgent need to dissect the molecular mechanisms driving the transition from benign adipose tissue expansion to pathological dysfunction [4].

Far from being an inert energy storage depot, adipose tissue is now recognized as a highly dynamic, metabolically active system central to whole-body energy homeostasis [5]. In response to chronic positive energy balance, a healthy adipose tissue mounts an adaptive response known as “healthy remodeling,” which involves both adipocyte hypertrophy (increased cell size) and hyperplasia (generation of new adipocytes from a pool of committed precursors) [6]. This process allows safe and efficient sequestration of excess lipids, preventing ectopic deposition in peripheral organs such as the liver and skeletal muscle. The capacity of adipose tissue to undergo this benign expansion, however, is finite and varies considerably between individuals, representing a critical determinant of metabolic health status [4].

When the limit of healthy expansion is surpassed, as occurs in the majority of individuals with obesity, the remodeling process becomes pathological. Rapid and excessive adipocyte hypertrophy outpaces the growth of the supporting vasculature, creating regions of local hypoxia due to an insufficient oxygen supply to enlarged adipocytes [7,8].

This hypoxic microenvironment serves as a primary cellular stressor, activating hypoxia-inducible factors (HIFs) that orchestrate a robust transcriptional response. A central consequence of this response is the induction of a chronic, low-grade state of inflammation within the adipose tissue, characterized by the upregulation of pro-inflammatory adipokines and potent chemoattractant signals [9,10]. These chemotactic cues drive the progressive recruitment and infiltration of circulating monocytes, which differentiate into adipose tissue macrophages (ATMs) and become a dominant source of additional inflammatory mediators [11,12]. This marked shift toward a pro-inflammatory secretory profile, orchestrated by both stressed adipocytes and infiltrating immune cells, is a defining hallmark of adipose tissue dysfunction and a fundamental pathogenic link to systemic insulin resistance [13].

At the cellular level, the failing adipose tissue is characterized not only by inflammation and the onset of fibrosis but also by a significant increase in adipocyte death [14]. The demise of hypertrophic, dysfunctional adipocytes is a key event that unleashes a vicious cycle of worsening tissue pathology. It is important to distinguish, however, between the physiological homeostatic turnover of adipocytes that occurs during normal tissue maintenance and the pathological cell death that characterizes the obese state. Basal adipocyte renewal is a tightly regulated process that does not trigger significant inflammation, as apoptotic cells are efficiently cleared by resident macrophages in an immunologically silent manner [4,8]. In obesity, the scale and nature of adipocyte death change substantially: the proportion of dying adipocytes increases markedly, and the predominant modes of death shift toward lytic, immunogenic forms, including necrosis and, as proposed in this review, ferroptosis, that actively recruit and activate pro-inflammatory macrophages [14,15]. The formation of crown-like structures around dead adipocytes provides direct histological evidence of this shift toward inflammation-driving cell death [14,15]. Adipocyte death in obesity is therefore best understood not as an exaggerated homeostatic process but as a qualitatively distinct pathological event driven by microenvironmental stresses such as hypoxia, mechanical strain, and metabolic overload [1,7,8].

Histologically, adipocyte death is visualized by the formation of crown-like structures (CLS), in which pro-inflammatory macrophages are found surrounding the lipid debris of necrotic-like adipocytes [14]. The extent of CLS infiltration correlates directly with the severity of obesity-related complications, including insulin resistance and the progression of NAFLD, in both rodent models and human patients [15,16]. This cell death-driven inflammation establishes a self-amplifying circuit: inflammatory mediators from macrophages further impair adipocyte function, promoting more death and attracting additional immune cell infiltration, thereby entrenching tissue dysfunction and systemic metabolic dysregulation.

Historically, adipocyte death in obesity has been viewed through the lens of passive necrosis or classical apoptosis. However, the landscape of regulated cell death has expanded substantially in the last decade, leading to the discovery and characterization of novel, molecularly defined death pathways. Among these, ferroptosis, an iron- and lipid peroxidation-dependent form of regulated necrosis first described by Dixon et al. in 2012, has emerged as a critical mechanism in a wide array of pathological conditions, including cancer, neurodegeneration, and ischemia–reperfusion injury [17,18,19]. Ferroptosis is fundamentally a metabolic form of death, intimately linked to intracellular iron handling, the availability of oxidizable polyunsaturated fatty acids (PUFAs) in cellular membranes, and the failure of antioxidant defense systems, most notably the glutathione (GSH)-dependent enzyme glutathione peroxidase 4 (GPX4) [20,21].

A growing body of evidence now points to the involvement of ferroptosis in the pathogenesis of metabolic diseases, including obesity. The dysregulated iron metabolism, heightened oxidative stress, and chronic inflammation that characterize the obese adipose tissue create a tissue microenvironment that is primed for ferroptotic execution [22,23].

Furthermore, the unique lipid profile of adipocytes, which are naturally enriched in lipid droplets and are sensitive to PUFA phospholipid remodeling, may render them particularly susceptible to the lipid peroxidation chain reaction that drives ferroptosis [24,25]. The involvement of ferroptosis in adipocytes not only represents a novel mechanism of cell loss but also triggers specific forms of cell-to-cell cross-talk. Recent findings demonstrate that signals released from ferroptotic adipocytes can powerfully shape the function and polarization of neighboring macrophages, thereby directly linking iron-dependent cell death to the propagation of metabolic inflammation [26]. Importantly, ferroptotic signaling in the adipose organ may exert context-dependent effects that extend beyond cell death. Recent evidence indicates that low-amplitude, sublethal ferroptotic signals can activate adaptive thermogenic programs, whereas excessive or chronic ferroptotic stress drives inflammation and tissue dysfunction [26]. This duality, protective signaling at low intensity versus pathological death at high intensity, is discussed in detail in Section 7.3 and represents a central theme for understanding the role of ferroptosis in obesity.

Given the centrality of iron dysregulation, redox imbalance, and lipid peroxidation in the etiology of ferroptosis, these pathways present attractive targets for therapeutic intervention. In this context, naturally derived bioactive compounds, or nutraceuticals, offer a promising avenue. Numerous plant-derived polyphenols and other phytochemicals have been shown to possess potent iron-chelating, antioxidant, and Nrf2-activating properties, all of which could potentially intercept the ferroptotic cascade at multiple levels [27,28]. Therefore, this review aims to critically synthesize the current molecular understanding of adipocyte ferroptosis within the context of obesity. We will provide a comprehensive overview of adipose tissue remodeling and its pathological limits, dissect the core molecular machinery of ferroptosis with a focus on its relevance to adipocyte biology, explore the emerging role of ferroptosis-mediated cross-talk between dying adipocytes and macrophages as a driver of metabolic inflammation, and finally, evaluate the therapeutic potential and molecular targets of select nutraceuticals capable of modulating ferroptosis pathways in the adipose tissue microenvironment.

## 2. Cellular and Molecular Mechanisms of Adipocyte Death in Obesity

The progressive metabolic deterioration observed in obesity is intimately linked to the cellular fate of adipocytes within the expanding adipose tissue. As the limits of healthy remodeling are surpassed, the hostile microenvironment, characterized by hypoxia, mechanical stress, and unresolved inflammation, precipitates a significant increase in adipocyte death. This cell loss is far from a passive consequence of tissue stress; rather, it functions as a critical pathological tipping point that actively drives disease progression by triggering immune cell recruitment, insulin resistance, and fibrotic remodeling [3,14]. Understanding the precise molecular pathways governing adipocyte demise is therefore essential for identifying novel therapeutic targets. Historically, the study of cell death in obesity has focused on classical apoptosis and necrosis; however, the last decade of research into regulated cell death has dramatically expanded this landscape, revealing a complex interplay of distinct yet partially overlapping death modalities within the dysfunctional adipose tissue.

The earliest mechanistic studies of adipocyte death in obesity identified classical apoptosis as a significant contributor. Apoptosis is a highly regulated, caspase-dependent process characterized by cell shrinkage, membrane blebbing, and the formation of apoptotic bodies that are, under homeostatic conditions, rapidly cleared by phagocytic macrophages in an immunologically silent manner [4,14]. In the obese adipose tissue, a multitude of cellular stressors, including hypoxia-induced endoplasmic reticulum stress, elevated concentrations of saturated free fatty acids, and high local levels of the pro-inflammatory cytokine TNF-α, can activate both the intrinsic mitochondrial and extrinsic death receptor pathways of apoptosis [1,2,14]. However, the massive scale of adipocyte death in severe obesity can overwhelm the phagocytic clearance capacity of resident macrophages, leading to secondary necrosis of uncleared apoptotic cells. This transition from silent to lytic cell death results in the release of intracellular contents, including cytotoxic lipids, ATP, and other damage-associated molecular patterns (DAMPs), which serve as potent pro-inflammatory signals and directly contribute to the propagation of metabolic inflammation.

A far more prominent feature in the histopathological landscape of obese adipose tissue is the hallmark of primary necrotic cell death. Histological examination of adipose tissue from both diet-induced obese mice and morbidly obese human subjects consistently reveals the presence of crown-like structures (CLS): aggregates of pro-inflammatory macrophages that surround the residual lipid droplets of large, necrotic adipocytes [14,15]. This form of death, traditionally viewed as passive and uncontrolled, is triggered when hypertrophic adipocytes are subjected to insurmountable mechanical stress on their plasma membrane and severe ATP depletion due to the combined effects of hypoxia and nutrient deprivation. The immediate and defining consequence of necrotic cell death is the unscheduled and complete release of cellular debris, lipids, and alarmins into the tissue interstitium. This acts as a profoundly chemotactic and activating stimulus for the innate immune system, establishing a self-reinforcing circuit of inflammation. The presence and density of CLS have been strongly correlated with the severity of systemic insulin resistance and the progression of NAFLD, establishing necrotic-like adipocyte death as a direct link between local tissue dysfunction and systemic metabolic disease [15,16].

Adding to this complexity, the highly inflammatory regulated cell death mode known as pyroptosis has also been mechanistically implicated in adipose tissue pathology. Pyroptosis is executed by caspase-1, which is typically activated following the assembly of cytosolic multiprotein complexes called inflammasomes, most notably NLRP3. In the context of obesity, numerous metabolic danger signals, including saturated fatty acids, ceramides, hyperglycemia, and extracellular ATP, can trigger NLRP3 inflammasome activation within both adipocytes and infiltrating macrophages [12,13]. Upon activation, caspase-1 not only cleaves gasdermin D, generating a pore-forming N-terminal fragment that causes cell swelling and osmotic lysis, but also proteolytically matures the potent pro-inflammatory cytokines interleukin (IL)-1β and IL-18 into their active, secreted forms. The lytic nature of pyroptotic death thus directly releases these master inflammatory cytokines along with other DAMPs into the extracellular milieu, powerfully amplifying the vicious cycle of metabolic inflammation and insulin resistance that defines the obese state. Furthermore, autophagy, an essential cellular homeostatic mechanism for the degradation and recycling of damaged organelles and protein aggregates, plays a dual and context-dependent role. While basal autophagy is required for normal adipocyte function and differentiation, its excessive or dysregulated activation by severe hypoxia and oxidative stress can trigger a morphologically distinct form of cell death termed autosis or autophagy-dependent cell death, providing yet another route to adipocyte loss. Crucially, specific selective autophagic processes are of paramount importance to the regulation of cell death beyond their direct role in cell suicide. Most notably, ferritinophagy, the selective autophagic degradation of the iron-storage protein ferritin mediated by the cargo receptor NCOA4, directly mobilizes intracellular iron stores, increasing the labile iron pool that is available to catalyze lethal lipid peroxidation reactions [29,30]. This positions selective autophagy as a critical upstream regulator of the most recently appreciated and fundamentally distinct form of adipocyte death: ferroptosis. The principal cell death modalities implicated in adipose tissue pathology are summarized in Table 1.

The seminal discovery of ferroptosis by Dixon and colleagues in 2012 introduced a transformative framework in cell death research, defining an iron-dependent and lipid peroxidation-driven form of regulated necrosis that is genetically, biochemically, and morphologically distinct from all other previously described death modalities [17]. Ferroptotic cells do not exhibit the characteristic features of apoptosis, such as cell shrinkage or caspase activation, nor the rapid swelling of necrosis, but are instead defined by a unique mitochondrial phenotype, shrunken mitochondria with increased membrane density, and the catastrophic, iron-catalyzed accumulation of lipid peroxides in cellular membranes that ultimately destroys membrane integrity [18,19]. The unique metabolic environment of the pathologically stressed adipocyte, characterized by an inherent sensitivity to lipid handling and storage, a potential for profound iron dysregulation, and a high background of oxidative stress, makes ferroptosis an exceptionally salient, yet until recently largely overlooked, mechanism driving adipocyte loss and adipose tissue dysfunction. The core molecular machinery that executes and defends against ferroptosis, and its specific pathological role within the obese adipose organ, will be the central focus of the subsequent section.

Summary of the key molecular features, immunogenic profiles, and histological signatures of the major regulated cell death pathways operating in the dysfunctional adipose tissue of obese individuals.

## 3. Ferroptosis in Adipose Tissue Dysfunction: Iron Metabolism, Lipid Peroxidation, GPX4, and Redox Imbalance

The unique metabolic profile of obese adipose tissue generates a cellular context that is uniquely favorable to ferroptosis. The convergence of dysregulated iron homeostasis, an abundance of oxidizable lipid substrates, and a compromised antioxidant defense network establishes a convergence of pro-ferroptotic conditions that lowers the threshold for ferroptosis execution in adipocytes. This section systematically dissects the core molecular machinery of ferroptosis, iron metabolism, lipid peroxidation, and the GPX4-glutathione axis, with a specific focus on their dysregulation in the context of adipose tissue dysfunction and obesity. A critical distinction must be made throughout this section between molecular mechanisms that have been directly demonstrated in adipocytes or adipose tissue and those that have been characterized primarily in other cell types or disease models. The core enzymatic machinery of ferroptosis, including the Fenton reaction, lipoxygenase activity, ACSL4/LPCAT3-dependent phospholipid remodeling, and the GPX4-glutathione system, has been dissected largely in cancer cell lines, fibroblasts, and non-adipose primary cells [17,18,20,24,31]. In adipocytes, direct evidence for dysregulation of these specific pathways is still emerging and derives from a more limited set of observations in the context of obesity [22,26,32,33]. Where the evidence derives from non-adipose systems, this is explicitly noted in the text. The reader should therefore consider the mechanistic framework presented herein as a synthesis of well-established ferroptosis biology that is increasingly, but not yet fully, corroborated in the adipose organ.

### 3.1. Iron Dysregulation in the Obese Adipose Tissue

Iron is an essential micronutrient that serves as a critical cofactor for numerous fundamental biological processes, including oxygen transport, mitochondrial respiration, and DNA synthesis. However, the redox-active nature of iron also renders it a potential hazard, as labile ferrous iron (Fe^2+^) can directly catalyze the Fenton reaction, converting hydrogen peroxide (H_2_O_2_) into the highly reactive hydroxyl radical (•OH), which initiates lipid peroxidation chain reactions [17,21]. The chemical basis of ferroptosis is rooted in this iron-dependent radical chemistry, and the regulation of lipid peroxidation by iron is a conserved vulnerability across diverse species, underscoring the fundamental biological importance of this death pathway [34]. Under physiological conditions, systemic and cellular iron homeostasis is tightly regulated by an elegant network of proteins controlling uptake, storage, and export. At the systemic level, the liver-derived peptide hormone hepcidin acts as the master regulator by binding to and inducing the degradation of ferroportin, the only known mammalian iron exporter, thereby controlling iron efflux from enterocytes, macrophages, and hepatocytes [35,36]. At the cellular level, iron regulatory proteins (IRPs) post-transcriptionally coordinate the expression of transferrin receptor 1 (TfR1) for iron uptake and ferritin for iron storage, ensuring that the intracellular labile iron pool remains within a safe range [37,38]. Beyond local iron handling, the mechanisms controlling cellular and systemic iron homeostasis are deeply interconnected, with the liver acting as a central sensor and distributor of iron via hepcidin, integrating signals from iron status, erythropoietic demand, and inflammation [39].

Compelling evidence indicates that obesity is a state of systemic and tissue-specific iron dysregulation. Clinical studies have consistently demonstrated elevated serum ferritin levels and altered iron distribution in adipose tissue of obese individuals, suggesting a pathological accumulation of iron within the adipose organ [32,33]. This adipose tissue iron overload is thought to arise from multiple sources. Adipocytes themselves exhibit increased expression of TfR1 and divalent metal transporter 1 (DMT1), facilitating enhanced iron uptake, while the expression of ferroportin appears to be decreased, impairing iron export [40,41]. Furthermore, within the obese adipose tissue, infiltrating pro-inflammatory macrophages undergo a phenotypic switch in their iron handling.

While alternatively activated (M2-like) macrophages are typically geared toward iron release via ferroportin, classically activated (M1-like) pro-inflammatory macrophages adopt a sequestration phenotype, characterized by high ferritin expression and low ferroportin levels, which retains iron at sites of inflammation [42,43]. This local iron trapping by inflammatory macrophages, in proximity to dying adipocytes, creates microenvironments with elevated catalytic iron availability that are primed for ferroptosis. The role of iron in adipose tissue is increasingly recognized as a silent pathogenic contributor in metabolic disease, with adipocyte iron overload directly contributing to insulin resistance, impaired adipokine secretion, and enhanced oxidative stress [33,41]. The resulting increase in the labile iron pool within adipocytes not only fuels the Fenton reaction but also promotes the activity of iron-dependent lipoxygenases (LOXs), enzymes that directly oxygenate polyunsaturated fatty acids in membrane phospholipids, initiating the lipid peroxidation cascade central to ferroptosis [20,25].

A further critical link between iron metabolism and ferroptosis sensitivity is the process of ferritinophagy. Ferritin is the primary intracellular iron storage protein, sequestering up to 4500 iron atoms in a redox-inert mineral core within a 24-subunit protein shell. The selective autophagic degradation of ferritin, mediated by the cargo receptor NCOA4, releases this stored iron into the labile pool, markedly increasing the availability of redox-active iron [29,44]. This process is tightly regulated by iron-dependent HERC2-mediated proteolysis of NCOA4, and is essential not only for erythropoiesis but also for the mobilization of iron stores under conditions of increased cellular demand or stress [44]. In conditions of metabolic stress, such as the oxidative environment of the obese adipose tissue, ferritinophagy can be upregulated, representing a source of free iron that directly sensitizes cells to ferroptosis [30]. Recent work has highlighted the importance of ferritinophagy at the intersection of iron metabolism and mitochondrial quality control in muscle aging, suggesting that this pathway may be a conserved mechanism of iron mobilization across tissues under metabolic stress [45]. While the regulation of NCOA4-mediated ferritinophagy in adipocytes remains an area of active investigation, its established role in mobilizing iron stores positions it as a likely key determinant of adipocyte ferroptosis susceptibility. Additionally, the process of glutaminolysis has been identified as a critical metabolic requirement for ferroptosis execution, as glutamine-derived α-ketoglutarate fuels the tricarboxylic acid cycle and sustains mitochondrial metabolism, which is necessary for the generation of reactive oxygen species that drive lipid peroxidation under cystine-deprived conditions [46]. Collectively, the convergence of increased iron uptake, reduced export, inflammatory macrophage-mediated iron sequestration, and potential ferritinophagy upregulation establishes a state of pathological iron overload in the adipose tissue of obese individuals, laying the essential groundwork for ferroptotic cell death.

### 3.2. Lipid Peroxidation: The Executioner of Ferroptosis

If iron dysregulation provides the spark, then lipid peroxidation is the fire that executes ferroptotic death. Ferroptosis is executed when the rate of lipid peroxide accumulation in cellular membranes overwhelms the capacity of intrinsic antioxidant systems to reduce them. Not all lipids are equally susceptible; the specificity of ferroptosis for certain polyunsaturated fatty acid (PUFA)-containing phospholipids (PUFA-PLs) is a defining feature of the process. Membranes enriched with arachidonic acid (AA, C20:4) and adrenic acid (AdA, C22:4) esterified into phosphatidylethanolamine (PE) are the preferred substrates for peroxidation and the most critical executioners of ferroptosis [24,25]. The incorporation of these highly oxidizable PUFAs into membrane phospholipids is not a random process but is actively catalyzed by specific enzymes, most notably acyl-CoA synthetase long-chain family member 4 (ACSL4) and lysophosphatidylcholine acyltransferase 3 (LPCAT3). ACSL4 ligates AA and AdA to coenzyme A, and LPCAT3 subsequently esterifies these activated fatty acids into membrane PE, thereby remodeling the cellular lipid landscape to create the very substrates whose peroxidation drives ferroptosis [24,31].

Consequently, the expression level and activity of ACSL4 are key determinants of cellular sensitivity to ferroptosis. The cell biology of ferroptosis is now understood to involve a complex interplay between organelle-specific lipid metabolism, membrane remodeling, and the spatial organization of pro- and anti-ferroptotic machinery, with the plasma membrane, endoplasmic reticulum, and mitochondria all contributing to the execution or prevention of ferroptotic death [31,47].

The peroxidation of these PUFA-PLs can proceed through two non-mutually exclusive mechanisms: enzymatic and non-enzymatic. The non-enzymatic, iron-dependent Fenton reaction generates hydroxyl radicals that abstract hydrogen atoms from the bis-allylic positions of PUFAs, initiating a self-propagating free radical chain reaction that generates lipid hydroperoxides (LOOHs). Simultaneously, the enzymatic pathway is driven by a family of iron-containing enzymes called lipoxygenases (LOXs), which directly catalyze the stereospecific oxygenation of free and esterified PUFAs [20,21]. The resulting lipid hydroperoxides, if not efficiently reduced to their corresponding inert lipid alcohols, can react with ferrous iron to generate lipid alkoxyl radicals, which in turn propagate further peroxidation, creating a catastrophic cascade that ultimately disrupts membrane integrity, increases permeability, and triggers cell death. Ferroptosis has now been mechanistically linked to a wide spectrum of human diseases, including not only cancer and neurodegeneration but also metabolic disorders, ischemic injury, and inflammation-driven pathologies, highlighting its broad pathophysiological relevance [19,48].

Adipocytes are intrinsically specialized for managing and storing large quantities of lipids. In obesity, the fatty acid composition of adipocyte membranes is altered, with changes in the ratio of saturated to unsaturated fatty acids that can impact the pool of available ferroptosis substrates. The metabolic determinants of ferroptosis sensitivity extend beyond the lipid composition of membranes to include the availability of specific nutrients and the activity of metabolic pathways that supply substrates for lipid peroxidation and antioxidant defense [49]. The expression and activity of ACSL4 and the abundance of AA/AdA-PE species in the adipocyte membrane represent critical, and as yet incompletely understood, determinants of ferroptosis sensitivity in this specific cell type.

The natural lipid-rich milieu of the adipocyte, combined with obesity-induced oxidative stress and iron overload, creates a context in which the threshold for initiating the lipid peroxidation cascade may be significantly lowered, rendering hypertrophic adipocytes especially vulnerable to this form of death [22,23]. Indeed, ferroptosis is increasingly viewed as a nexus connecting cellular metabolism, redox biology, and disease pathogenesis, with the metabolic syndrome and its complications representing a particularly relevant context for ferroptosis-driven pathology [50,51].

### 3.3. The GPX4-Glutathione System: The Principal Guardian Against Ferroptosis

The single most critical defense against the execution of ferroptosis is the selenoenzyme glutathione peroxidase 4 (GPX4). GPX4 is the only mammalian enzyme capable of directly reducing toxic lipid hydroperoxides (LOOHs) within biological membranes to their corresponding non-toxic lipid alcohols (LOHs), thereby breaking the chain reaction of lipid peroxidation at its source [20,52]. The catalytic cycle of GPX4 requires the tripeptide glutathione (GSH) as an obligate electron donor; oxidized GSH (GSSG) is then recycled back to its reduced form by glutathione reductase using NADPH. Consequently, the functional integrity of the GPX4-GSH axis is absolutely dependent on the availability of intracellular cysteine, the rate-limiting precursor for GSH synthesis.

Cysteine is imported into cells primarily via the cystine/glutamate antiporter system xc^−^, a heterodimeric transporter composed of the light chain subunit SLC7A11 (xCT) and the heavy chain subunit SLC3A2. By importing cystine (the oxidized dimeric form of cysteine) in exchange for glutamate, system xc^−^ maintains the intracellular cysteine pool necessary for GSH biosynthesis. The genetic or pharmacological inactivation of GPX4 results in the unrestrained accumulation of lipid peroxides and rapid ferroptotic cell death, a phenotype that can be rescued by lipophilic radical-trapping antioxidants (RTAs) such as ferrostatin-1 and liproxstatin-1 [17,53]. Similarly, inhibition of system xc^−^ by erastin depletes intracellular GSH, indirectly inactivating GPX4 and triggering ferroptosis [17,18].

The regulation of lipid peroxidation and ferroptosis is a deeply conserved biological process, with homologous pathways identified in organisms ranging from plants to mammals, indicating that the core machinery of ferroptosis represents an ancient mechanism of cellular demise [34].

In the context of obesity, the GPX4-GSH defense network is under significant siege. The chronic, low-grade systemic and local inflammation characteristic of obesity generates a persistent oxidative burden that can deplete GSH stores. Furthermore, the hypoxic adipose tissue microenvironment can transcriptionally and post-translationally modulate the expression of GPX4 and SLC7A11, potentially weakening this critical defense axis [51,54]. A reduction in GPX4 expression or activity, or a limitation in cysteine/GSH availability, would profoundly sensitize adipocytes to the lethal lipid peroxidation driven by the concurrent iron overload and PUFA-PL enrichment described above. The role of mitochondria in ferroptosis adds a layer of complexity to this regulatory network. While the canonical ferroptosis pathway can proceed independently of mitochondrial function, mitochondria play a modulatory role through their involvement in lipid metabolism, ROS production, and the provision of substrates for the tricarboxylic acid cycle that sustain ferroptosis under specific conditions [55]. The delicate balance between pro-ferroptotic forces (iron, PUFA-PLs, LOXs, mitochondrial metabolism) and the anti-ferroptotic GPX4-GSH system is a decisive determinant of adipocyte fate.

### 3.4. Parallel and Complementary Defense Systems

The centrality of the GPX4-GSH axis does not preclude the existence of additional, genetically encoded mechanisms that guard against ferroptosis, and their discovery has significantly deepened our understanding of this cell death pathway. Notably, ferroptosis suppressor protein 1 (FSP1), previously known as apoptosis-inducing factor mitochondrial 2 (AIFM2), was identified as a parallel ferroptosis defense system that operates independently of glutathione [56,57]. FSP1 is a flavoprotein that is myristoylated and targeted to the plasma membrane, where it functions as an NAD(P)H-dependent oxidoreductase capable of reducing coenzyme Q_10_ (CoQ_10_, ubiquinone) to its potent lipophilic radical-trapping antioxidant form, ubiquinol (CoQ_10_H_2_). This reduced form of CoQ_10_ directly intercepts lipid peroxyl radicals, thereby halting the peroxidation chain reaction in a manner analogous to GPX4 but using a distinct, non-protein thiol-dependent mechanism. The expression and activity of FSP1 in adipocytes under conditions of obesity remain largely unexplored but represent a potentially critical variable in determining ferroptosis susceptibility, especially in contexts where the GPX4-GSH axis is compromised.

Additionally, other pathways contribute to the cellular ferroptosis defense network, including the GTP cyclohydrolase 1 (GCH1)-tetrahydrobiopterin (BH4) pathway, which can remodel the lipid membrane to a less oxidizable state and generate lipophilic radical-trapping antioxidant species [58].

### 3.5. Nrf2: The Master Transcriptional Orchestrator of Anti-Ferroptotic Defenses

The transcription factor Nuclear factor erythroid 2-related factor 2 (Nrf2) serves as the principal transcriptional coordinator of the cellular antioxidant and cytoprotective response, playing a pivotal role in regulating resistance to ferroptosis. Under basal conditions, Nrf2 is constitutively targeted for proteasomal degradation through its interaction with the cysteine-rich adaptor protein Keap1 (Kelch-like ECH-associated protein 1), which acts as a substrate for the Cullin-3 E3 ubiquitin ligase complex [59,60]. In response to oxidative or electrophilic stress, specific cysteine residues within Keap1 are chemically modified, causing a conformational change that disrupts Nrf2 ubiquitination and degradation. Newly stabilized and accumulated Nrf2 translocates to the nucleus, where it heterodimerizes with small Maf proteins and binds to Antioxidant Response Elements (ARE) in the promoter regions of its target genes, activating a broad transcriptional program [61,62].

Critically, many genes that are central to ferroptosis resistance are direct Nrf2 targets. These include genes involved in GSH synthesis and regeneration (the catalytic and modulatory subunits of glutamate-cysteine ligase, GCLC and GCLM), the cystine transporter (SLC7A11), the antioxidant enzyme GPX4 itself, and proteins involved in iron sequestration and export (ferritin heavy and light chains, FTH1 and FTL, and ferroportin) [63,64].

By coordinately upregulating these key defense systems, Nrf2 activation can powerfully elevate the cellular threshold for ferroptosis induction.

Conversely, a failure of Nrf2 signaling, as may occur in states of chronic oxidative stress or aging, would be predicted to increase ferroptosis susceptibility. In the obese adipose tissue, the Nrf2 pathway is activated as a compensatory response to oxidative stress, but its activity may be insufficient to counteract the overwhelming pro-ferroptotic stimuli, contributing to the net sensitization of adipocytes to death [50,65]. The Nrf2 pathway therefore represents a central node at which multiple upstream signals converge to determine ferroptotic fate and constitutes one of the most attractive molecular targets for nutraceutical intervention, as will be discussed in detail in the subsequent sections. The key molecular alterations that render obese adipose tissue susceptible to ferroptosis are summarized in Table 2.

Overview of the core pathway components, their pro-ferroptotic alterations in obesity, and the resulting consequences for adipocyte ferroptosis susceptibility.

## 4. Ferroptosis-Mediated Cell-to-Cell Cross-Talk: Adipocyte–Macrophage Interactions and Metabolic Inflammation

Cell death within the adipose tissue is not a terminal, isolated event but rather the initiating signal for a complex cascade of intercellular communication that fundamentally shapes the tissue microenvironment and ultimately dictates systemic metabolic outcomes.

The recognition that adipocyte death is a primary trigger for the massive macrophage infiltration and activation characteristic of obese adipose tissue is now well established [10,11,14]. A defining histological hallmark of dysfunctional adipose tissue is the progressive accumulation of macrophages, which can constitute up to 40–50% of the stromal vascular fraction in morbid obesity, compared to approximately 5–10% in the lean, healthy state. This recruitment is driven by a diverse repertoire of chemotactic signals released from stressed, hypertrophic, and dying adipocytes, including monocyte chemoattractant protein-1 (MCP-1/CCL2), leukotriene B4, and various damage-associated molecular patterns (DAMPs) [12,13]. Importantly, the biological function of these recruited macrophages is not monolithic; their polarization state critically dictates their impact on tissue homeostasis. Adipose tissue macrophages are a remarkably heterogeneous population whose biology and properties span a spectrum of activation states beyond the simplified M1/M2 dichotomy, with their functional phenotype being dynamically shaped by signals emanating from the local tissue microenvironment, including those released by dying adipocytes [67]. In the lean state, resident adipose tissue macrophages (ATMs) predominantly exhibit an alternatively activated, anti-inflammatory phenotype (often termed M2-like), characterized by the secretion of IL-10 and the expression of arginase-1 and CD206, thereby contributing to tissue repair, extracellular matrix remodeling, and the maintenance of insulin sensitivity. Obesity, however, drives a marked and phenotypically profound shift in ATM polarization toward a classically activated, pro-inflammatory state (M1-like), marked by the robust secretion of TNF-α, IL-6, and IL-1β, and the surface expression of CD11c. This M1-like macrophage population actively promotes local inflammation, adipocyte insulin resistance, and the progression of fibrosis [12,13,68]. The molecular signals that govern this switch from an anti-inflammatory to a pro-inflammatory ATM phenotype have been intensely investigated, with the secretome of dying adipocytes emerging as a principal driver.

The discovery of ferroptosis as a distinct, immunogenic form of regulated necrosis has prompted a critical re-evaluation of the nature of the adipocyte death secretome and its specific consequences for macrophage biology. The fundamental question is whether ferroptosis, as a specific mode of cell death, elicits an immunogenic profile that is distinct from classical apoptosis or primary necrosis. While apoptosis is exquisitely designed to be immunologically silent, with the ordered packaging of cellular contents into apoptotic bodies for rapid phagocytic clearance, ferroptosis, like other lytic forms of regulated necrosis, results in the catastrophic rupture of the plasma membrane and the unscheduled release of a complex and bioactive mixture of intracellular contents into the tissue interstitium. The molecular composition of this “ferroptotic secretome” is uniquely shaped by the biochemical processes that define ferroptotic death itself. It includes classical DAMPs such as high mobility group box 1 (HMGB1), ATP, and heat shock proteins, but also, critically, a unique and pathognomonic spectrum of oxidized lipid mediators and electrophilic species generated directly by the lipid peroxidation cascade that executes the cell [18,19,69]. The release of extensively oxidized phospholipids and reactive lipid electrophiles, such as 4-hydroxynonenal (4-HNE) and malondialdehyde (MDA), constitutes a signature molecular fingerprint of ferroptotic death. These lipid-derived species are not merely inert byproducts of membrane destruction; they are potent signaling molecules capable of forming covalent adducts with proteins, activating pattern recognition receptors, and directly triggering inflammatory signaling cascades in neighboring cells [19,65].

The presence of this unique cocktail of DAMPs and lipid mediators in the ferroptotic secretome suggests that ferroptotic adipocyte death may act as a uniquely potent and qualitatively distinct activator of the innate immune system within the obese adipose tissue.

A recent study by Wang and colleagues has provided the most direct evidence to date that adipocyte-derived ferroptotic signaling may play a causal role in shaping the immunological landscape of obese adipose tissue [26]. While these findings are compelling, they derive from a single model system, and independent replication will be important to confirm their generalizability. Their work elegantly demonstrated that in the context of diet-induced obesity, ferroptosis actively occurs in adipocytes, and that the complex secretome released by these ferroptotic cells is not merely generically pro-inflammatory, but rather actively and specifically instructs the polarization of infiltrating macrophages toward a distinct pro-inflammatory state that directly exacerbates metabolic disease. The study showed, through a series of genetic and pharmacological loss- and gain-of-function experiments, that pro-ferroptotic signals emanating from dying adipocytes are sufficient to promote the accumulation of macrophages and to drive their polarization toward an M1-like phenotype within the adipose tissue. This ferroptosis-driven shift in ATM polarization directly impairs adipocyte insulin signaling and promotes ectopic lipid accumulation in peripheral organs. Conversely, genetic or pharmacological inhibition of adipocyte ferroptosis was sufficient to significantly mitigate macrophage infiltration and to rebalance their polarization away from a pro-inflammatory state, thereby improving systemic glucose homeostasis and reducing the severity of obesity-driven metabolic pathology [26]. These findings establish a direct, causal, and bidirectional pathogenic axis: obesity-induced metabolic stress triggers adipocyte ferroptosis, and the consequent ferroptotic secretome drives a unique program of macrophage-mediated inflammation, which in turn further stresses and sensitizes surviving adipocytes, perpetuating a self-amplifying cycle of tissue dysfunction and metabolic decline.

The centrality of iron to the execution of ferroptosis adds a particularly intricate layer of complexity to this cellular cross-talk, as adipose tissue macrophages are not merely passive sensors of ferroptotic death but are themselves active and dynamic participants in the local regulation of iron homeostasis that can either promote or protect against ferroptosis in neighboring adipocytes. The distinct macrophage polarization states are functionally coupled to profoundly different cellular iron-handling phenotypes. As introduced previously, classically activated M1-polarized, pro-inflammatory macrophages characteristically upregulate ferritin expression and downregulate the iron exporter ferroportin, thereby adopting an iron-sequestering profile that retains large quantities of iron within the macrophage [42,43]. Macrophages are central to systemic iron handling, functioning as the primary recyclers of iron from senescent erythrocytes and damaged cells; this process requires transient macrophages in the liver that efficiently recover and release iron back into the circulation under physiological conditions [70]. However, in the chronic inflammatory milieu of obese adipose tissue, this homeostatic iron recycling function is subverted, and macrophages instead adopt a sequestration phenotype that traps iron locally [71]. While this sequestration strategy may serve a beneficial bacteriostatic function during acute infection by limiting iron availability to extracellular pathogens, in the context of chronic, sterile metabolic inflammation within obese adipose tissue, it has pathological consequences.

The progressive accumulation of iron-laden, M1-like ATMs creates localized microenvironments with an exceptionally high catalytic iron potential, particularly concentrated in macrophage clusters such as the crown-like structures (CLS) surrounding dying adipocytes [66,71]. The iron content of adipose tissue macrophages is significantly altered in obesity, with increased iron loading of ATMs contributing to their pro-inflammatory polarization and creating a feed-forward loop in which iron-rich, inflamed macrophages release cytokines that further dysregulate iron handling in neighboring adipocytes [33,66].

Furthermore, the eventual death and turnover of these iron-loaded macrophages would result in the concentrated, localized release of vast amounts of redox-active iron directly onto adjacent, surviving adipocytes. This macrophage-derived iron bolus would effectively provide the fuel for iron-catalyzed Fenton chemistry and lipid peroxidation, powerfully sensitizing the neighboring adipocytes to ferroptotic death. In addition to this direct iron-dumping mechanism, the pro-inflammatory cytokines secreted by M1-like macrophages, particularly TNF-α, can directly modulate the expression of iron-regulatory proteins in target cells, promoting iron uptake through increased transferrin receptor expression and potentially increasing the labile iron pool in surviving adipocytes [37,67].

This establishes a dynamic, bidirectional, and self-amplifying pathogenic circuit: adipocyte ferroptosis polarizes macrophages toward an M1-like, iron-sequestering phenotype, and these iron-laden, pro-inflammatory macrophages, through cytokine secretion and eventual iron release, reciprocally sensitize the surviving adipocyte population to ferroptosis, thereby entrenching and propagating the cycle of death and inflammation within the obese adipose tissue.

## 5. Consequences of Adipocyte Ferroptosis: Neuroimmune Crosstalk and Multi-Organ Complications

The pathogenic consequences of adipocyte ferroptosis extend far beyond the local adipose tissue microenvironment, engaging systemic inflammatory pathways that contribute to the multi-organ complications characteristic of advanced obesity. Importantly, ferroptosis has been shown to increase obesity through a novel crosstalk mechanism between adipocytes and the neuroimmune system, wherein ferroptotic signals from the adipose tissue can engage systemic inflammatory pathways that involve neuroimmune activation, thereby connecting local adipocyte death to central nervous system inflammation and whole-body energy dysregulation [72]. This local adipocyte–macrophage ferroptotic loop may represent the initiating event in a broader systemic inflammatory cascade, as ferroptosis-derived DAMPs and lipid mediators released from the adipose tissue can enter the circulation and engage neuroimmune pathways, creating an adipose–brain inflammatory axis that further dysregulates energy homeostasis and promotes obesity progression [72]. The activation of microglia and the recruitment of peripheral immune cells to the hypothalamus, a key region for the regulation of energy balance, could further dysregulate appetite control, energy expenditure, and glucose homeostasis, creating a self-perpetuating loop that exacerbates obesity.

**Figure 1:**
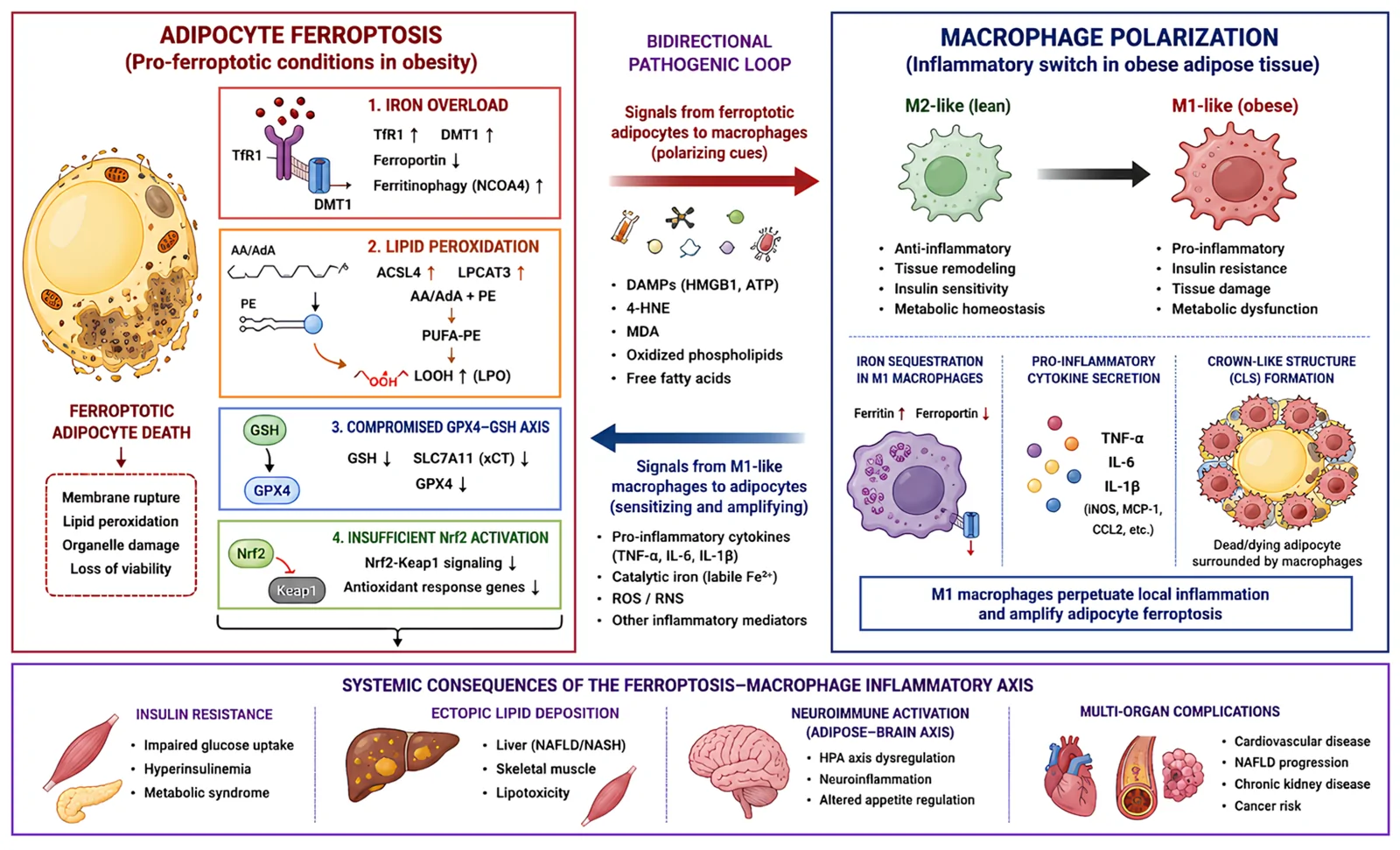
The adipocyte ferroptosis–macrophage inflammatory axis in obesity. Schematic representation of the bidirectional pathogenic loop linking ferroptotic adipocyte death to macrophage-driven metabolic inflammation in the obese adipose tissue. Pro-ferroptotic conditions (iron overload, ACSL4-mediated phospholipid remodeling, GPX4-GSH axis compromise, and insufficient Nrf2 activation) drive adipocyte ferroptosis, releasing a distinct secretome of damage-associated molecular patterns (DAMPs) and oxidized lipid mediators (4-HNE, MDA). These signals, rather than pre-formed cytokines, may act as potent polarizing cues that promote the polarization of infiltrating macrophages toward a pro-inflammatory M1-like phenotype. These M1-polarized macrophages are characterized by iron sequestration and the secretion of pro-inflammatory cytokines (TNF-α, IL-6, IL-1β). In turn, iron-laden macrophages release catalytic iron and inflammatory mediators that further sensitize surviving adipocytes to ferroptotic death, establishing a self-amplifying circuit. This local pathogenic loop propagates systemic consequences, including insulin resistance, ectopic lipid deposition, neuroimmune activation via the adipose–brain axis, and multi-organ complications. Abbreviations: 4-HNE, 4-hydroxynonenal; AA, arachidonic acid; ACSL4, acyl-CoA synthetase long-chain family member 4; AdA, adrenic acid; ATMs, adipose tissue macrophages; CLS, crown-like structures; DAMPs, damage-associated molecular patterns; DMT1, divalent metal transporter 1; GPX4, glutathione peroxidase 4; GSH, glutathione; LPCAT3, lysophosphatidylcholine acyltransferase 3; LOOH, lipid hydroperoxides; LPO, lipid peroxidation; MDA, malondialdehyde; NCOA4, nuclear receptor coactivator 4; Nrf2, nuclear factor erythroid 2-related factor 2; PE, phosphatidylethanolamine; TfR1, transferrin receptor 1.

The interplay between adipocyte ferroptosis and the systemic inflammatory response may also have far-reaching consequences beyond the adipose organ and the central nervous system. Notably, recent work has highlighted how the adipokine leptin, whose circulating levels are markedly elevated in obesity, can synergize with the chemokines CCL3 and CCL4, both of which are produced by activated macrophages and stressed adipocytes, to promote neuroinflammation and white matter degeneration in models of NAFLD [73]. This underscores the principle that the inflammatory signals originating from the ferroptotic adipocyte–macrophage interface may not only drive local tissue dysfunction and systemic insulin resistance but could also contribute to the multi-organ complications of obesity, including hepatic and neurological pathology, via the systemic dissemination of pro-inflammatory and chemotactic mediators released into the circulation. The emerging concept of ferroptosis-mediated crosstalk between adipocytes and the neuroimmune system provides a new mechanistic framework for understanding how adipose tissue dysfunction can drive neuroinflammation, cognitive decline, and central dysregulation of energy balance in obesity [72]. Furthermore, the systemic iron dysregulation of obesity, with its associated elevation in serum ferritin and altered hepcidin levels, could impact ferroptosis sensitivity in distant organs, including the liver, heart, and kidney, potentially contributing to the multi-organ complications of the metabolic syndrome [32,39,51]. Ferroptosis has been mechanistically linked to the pathogenesis of adiposity-based chronic disease (ABCD), a term that encompasses the full spectrum of obesity-related complications, including type 2 diabetes, NAFLD, cardiovascular disease, and certain cancers, highlighting the potential for ferroptosis-targeted interventions to address multiple obesity-related conditions simultaneously [23]. The hepatokine-adipokine-immune axis, in which the liver-derived hormone hepcidin and adipocyte-derived signals such as leptin converge to regulate systemic iron homeostasis and inflammation, represents a particularly important area for future research [36,73]. This putative adipocyte–brain inflammatory axis, potentially fueled by ferroptotic death in the adipose organ, and its ripple effects across multiple organ systems, represents an emerging area for future investigation and could reveal new therapeutic targets for both the metabolic and neurological complications of obesity. However, it should be noted that the evidence supporting a direct ferroptosis–neuroimmune axis in obesity is still in its infancy, being based on a limited number of preclinical studies, and requires independent replication and validation before firm mechanistic conclusions can be drawn. The bidirectional pathogenic loop linking adipocyte ferroptosis to macrophage-driven inflammation and its systemic consequences is illustrated in Figure 1.

## 6. Nutraceutical Modulation of Adipocyte Ferroptosis: Polyphenols and Other Bioactive Compounds Targeting Nrf2, GPX4, Iron Homeostasis, and Oxidative Stress Pathways

The emerging recognition of ferroptosis as a potential contributor to adipocyte death and adipose tissue inflammation in obesity has opened new therapeutic horizons. The molecular machinery of ferroptosis, spanning iron dysregulation, lipid peroxidation, and the failure of intrinsic antioxidant defenses, presents multiple nodes that are pharmacologically targetable. In this context, naturally derived bioactive compounds, or nutraceuticals, offer a particularly attractive and promising strategy. Many plant-derived polyphenols and other phytochemicals have evolved to interact with precisely these pathways, possessing potent iron-chelating, radical-trapping antioxidant, and Nrf2-activating properties [27,28]. Unlike synthetic inhibitors of ferroptosis, such as ferrostatin-1 or liproxstatin-1, which are primarily research tools, many nutraceuticals have a long history of safe human consumption and are supported by a growing body of clinical evidence for metabolic benefits. A comprehensive review of natural products for the treatment of NAFLD has highlighted the remarkable diversity of phytochemicals with hepatoprotective and metabolic benefits, many of which act through mechanisms directly relevant to ferroptosis modulation, including the activation of Nrf2, the inhibition of lipid peroxidation, and the reduction in inflammatory signaling [74]. This section systematically reviews key nutraceuticals with the potential to intercept the ferroptotic cascade at distinct levels, focusing on their molecular mechanisms of action with relevance to adipocyte biology and adipose tissue dysfunction in obesity. It must be emphasized at the outset that direct experimental evidence for nutraceutical-mediated inhibition of ferroptosis specifically in adipocytes within obese adipose tissue remains lacking. The studies discussed in this section derive primarily from (1) in vitro models of ferroptosis in non-adipose cell types; (2) in vivo models of metabolic disease in which ferroptosis markers have been assessed in liver, kidney, brain, or other tissues; (3) the well-characterized iron-chelating, radical-trapping antioxidant, and Nrf2-activating properties of these compounds established in cell-free and cell-based assays. Their efficacy in suppressing adipocyte ferroptosis in the obese adipose microenvironment therefore represents a biologically plausible but as yet unproven hypothesis requiring dedicated experimental validation.

To further clarify the level of evidence, it is useful to classify the compounds discussed below into three categories: (i) compounds with demonstrated anti-ferroptotic activity in multiple non-adipose cell types and in vivo metabolic disease models (e.g., curcumin, resveratrol, quercetin, sulforaphane); (ii) compounds with demonstrated metabolic benefits in adipose tissue in vivo but without direct ferroptosis-specific readouts (e.g., BPF and its combination with Cynara cardunculus); and (iii) compounds for which anti-ferroptotic activity in adipocytes remains entirely untested (e.g., astaxanthin). This classification is intended to guide the reader in assessing the strength of evidence for each intervention.

**Figure 2:**
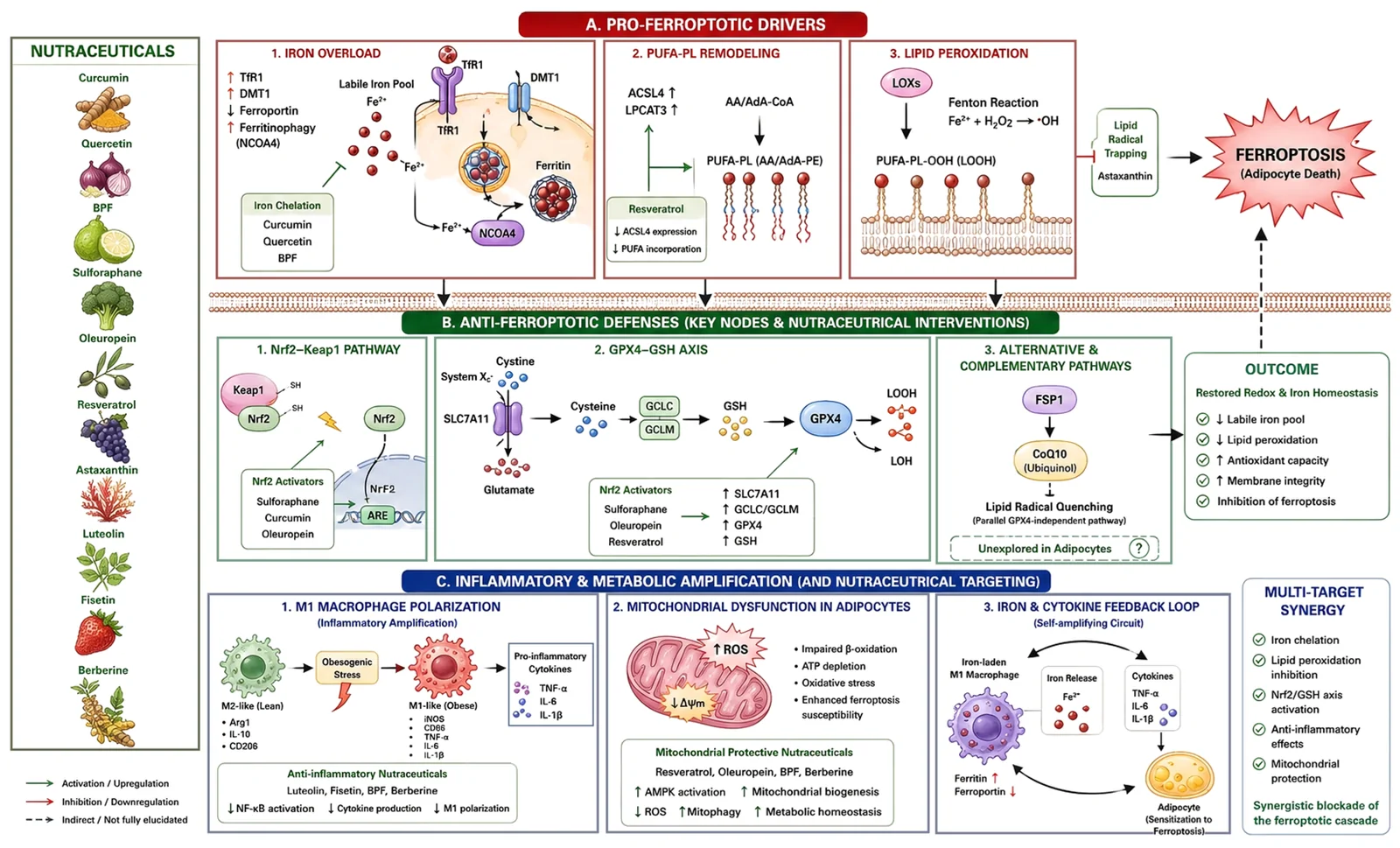
Nutraceutical targeting of the ferroptosis cascade in adipocytes. Schematic overview of the molecular nodes governing ferroptosis execution and defense, illustrating the points of intervention by plant-derived nutraceuticals. Compounds with iron-chelating properties (curcumin, quercetin, BPF) reduce the labile iron pool available for Fenton chemistry. Lipophilic radical-trapping antioxidants (astaxanthin) intercept lipid peroxyl radicals at the membrane level. Nrf2-activating compounds (sulforaphane, curcumin, oleuropein, resveratrol) upregulate the GPX4-GSH axis and iron-regulatory genes. Anti-inflammatory agents (luteolin, fisetin, BPF, berberine) counteract M1-macrophage-driven inflammatory amplification. Mitochondrial protective compounds (resveratrol, oleuropein, BPF, berberine) preserve metabolic homeostasis. Collectively, these multi-targeted interventions offer the potential for synergistic blockade of the ferroptotic cascade. Abbreviations: ACSL4, acyl-CoA synthetase long-chain family member 4; AMPK, AMP-activated protein kinase; BPF, bergamot polyphenolic fraction; CoQ_10_, coenzyme Q_10_; FSP1, ferroptosis suppressor protein 1; GCLC/GCLM, glutamate-cysteine ligase catalytic/modulatory subunits; GPX4, glutathione peroxidase 4; GSH, glutathione; Keap1, Kelch-like ECH-associated protein 1; LOOH, lipid hydroperoxides; LOX, lipoxygenase; LPCAT3, lysophosphatidylcholine acyltransferase 3; Nrf2, nuclear factor erythroid 2-related factor 2; PUFA-PL, polyunsaturated fatty acid-containing phospholipid; SLC7A11, solute carrier family 7 member 11.

At the molecular level, the nutraceuticals discussed in this section can in principle protect adipocytes from ferroptosis through four complementary mechanisms. First, iron chelation reduces the labile iron pool available to catalyze the Fenton reaction, thereby limiting the initiation of lipid peroxidation; compounds such as curcumin, quercetin, and BPF possess this property [27,75,76,77]. Second, direct radical trapping at the membrane level, exemplified by astaxanthin, which spans the lipid bilayer, intercepts propagating lipid peroxyl radicals and halts the chain reaction [28,78]. Third, Nrf2 activation upregulates a coordinated battery of ferroptosis-defense genes, including GPX4, SLC7A11, GCLC/GCLM, ferritin, and ferroportin, thereby simultaneously enhancing lipid peroxide detoxification, glutathione synthesis, and iron sequestration; sulforaphane is the most potent natural Nrf2 activator in this class, with curcumin, oleuropein, and resveratrol also demonstrating robust activity [60,62,79,80,81,82]. Fourth, AMPK activation, exhibited by BPF and berberine, promotes metabolic reprogramming that supports mitochondrial homeostasis and limits the excessive ROS production that can otherwise lower the ferroptotic threshold [77,83]. The molecular rationale for each nutraceutical is summarized in Figure 2. It must be reiterated, however, that these mechanisms have been demonstrated primarily in non-adipose cell types and disease models; whether they translate into effective suppression of adipocyte ferroptosis within the in vivo obese adipose microenvironment remains to be experimentally established.

### 6.1. Polyphenols as Multi-Targeted Ferroptosis Modulators

Polyphenols constitute a vast and structurally diverse family of plant secondary metabolites, ubiquitously present in fruits, vegetables, tea, coffee, and extra virgin olive oil, and are increasingly recognized for their pleiotropic health-promoting effects [84,85]. Their chemical structure, characterized by one or more aromatic rings bearing hydroxyl groups, directly endows many polyphenols with intrinsic radical-scavenging and metal-chelating capabilities, making them conceptually ideal candidates for intercepting the radical-driven chain reaction of lipid peroxidation that defines ferroptosis [27]. However, their biological effects extend far beyond direct antioxidant activity, as they can modulate intracellular signaling pathways and gene expression programs central to ferroptosis defense. The potential of natural antioxidants, including polyphenols, to counteract endothelial dysfunction and vascular inflammation, pathologies that share common oxidative and inflammatory mechanisms with adipose tissue dysfunction, further supports their therapeutic relevance in the context of obesity-related metabolic diseases [86].

Among the most extensively studied polyphenols, curcumin, the principal curcuminoid derived from turmeric (*Curcuma longa*), exhibits a remarkably broad spectrum of molecular targets that converge on ferroptosis-relevant pathways. Curcumin is a potent activator of the Nrf2-Keap1 pathway, upregulating a battery of antioxidant and cytoprotective genes, including those involved in GSH synthesis and iron sequestration [79,87]. Simultaneously, curcumin can directly chelate iron, reducing the labile iron pool available for Fenton chemistry, and functions as a direct scavenger of lipid-derived free radicals, thereby potentially intercepting the lipid peroxidation cascade at multiple levels [75]. In the context of obesity, curcumin supplementation has been shown to ameliorate systemic inflammation and improve insulin sensitivity, effects that may be partly mediated by the protection of adipocytes from oxidative stress-induced death [88]. The effects of curcumin on human health extend to the modulation of multiple signaling pathways, including NF-κB, STAT3, and PPARγ, all of which intersect with adipocyte biology, inflammation, and cell survival, further supporting its potential as a multi-targeted modulator of ferroptosis in the adipose tissue [79,87]. Similarly, resveratrol, a stilbenoid found in grapes and red wine, has demonstrated the capacity to protect against ferroptosis in multiple cellular contexts. Resveratrol activates Nrf2, upregulates GPX4 expression, and preserves mitochondrial function, thereby fortifying the cellular anti-ferroptotic defense network [80,89]. A systematic review of the molecular alterations in ferroptosis and the effects of resveratrol has confirmed that this polyphenol can modulate key ferroptosis regulators, including GPX4, SLC7A11, and ACSL4, across diverse cellular models, providing robust evidence for its anti-ferroptotic activity [80]. The flavonoid quercetin, abundant in capers, onions, and berries, has also been specifically implicated in ferroptosis modulation. Quercetin can inhibit lipid peroxidation, activate the Nrf2/HO-1/GPX4 signaling axis, and chelate iron, and has been shown to protect against ferroptosis in various models of tissue injury [76,90].

The specific interaction between quercetin and ferroptosis has been the subject of dedicated investigation, demonstrating that quercetin can protect cells from iron-dependent lipid peroxidation-driven death by upregulating GPX4, reducing intracellular iron accumulation, and directly scavenging lipid peroxyl radicals [76]. Fisetin, another naturally occurring flavonoid found in strawberries and apples, shares these pleiotropic ferroptosis-modulating properties, acting as both a direct antioxidant and a signaling molecule that can upregulate Nrf2-dependent defenses, with particular relevance to metabolic disorders [91]. Luteolin, a flavone present in celery, parsley, and artichoke, has demonstrated potent anti-inflammatory and antioxidant activities, with the ability to suppress pro-inflammatory cytokine production and inhibit oxidative stress, properties that would be beneficial in counteracting the inflammatory milieu that sensitizes adipocytes to ferroptosis [92].

### 6.2. Bergamot Polyphenolic Fraction: A Multi-Targeted Metabolic Nutraceutical

A nutraceutical of particular interest in the context of metabolic diseases is the bergamot polyphenolic fraction (BPF), derived from the endemic Calabrian citrus fruit *Citrus bergamia*. BPF is characterized by a unique and rich profile of flavonoids, including neoeriocitrin, naringin, and neohesperidin, and has been demonstrated in numerous preclinical and clinical studies to exert potent lipid-lowering, anti-inflammatory, and antioxidant effects [77,93]. The molecular mechanisms of BPF have been shown to involve activation of AMP-activated protein kinase (AMPK) and the modulation of cellular redox status, both of which are functionally coupled to ferroptosis regulation. The effect of natural antioxidants in the development of metabolic syndrome, with a specific focus on BPF, has been systematically reviewed, demonstrating that BPF can improve dyslipidemia, reduce systemic inflammation, and ameliorate insulin resistance, effects that are consistent with the protection of metabolic tissues from oxidative stress-induced damage [77]. In a mouse model of NAFLD, BPF not only improved dyslipidemia and reduced hepatic steatosis but also attenuated systemic and tissue-level oxidative stress and inflammation [93].

Notably, recent evidence from our laboratories has demonstrated that an innovative combination of BPF and *Cynara cardunculus* L. extract reduced weight gain and promoted the browning of white adipose tissue in obese mice, indicating a direct beneficial effect on the adipose organ that could partly stem from the protection of adipocytes from stress-induced death [94]. This same combination has demonstrated synergistic phytochemical properties in models of NAFLD, where the BPF and *Cynara cardunculus* extract acted through complementary mechanisms to reduce hepatic lipid accumulation, oxidative stress, and inflammatory cytokine production, providing a paradigm for how rationally designed nutraceutical combinations can achieve superior therapeutic effects compared to single agents [95]. The mechanisms of action of these natural extracts are consistent with the modulation of ferroptosis-relevant pathways, including the upregulation of Nrf2-dependent antioxidant defenses and the preservation of mitochondrial function [94,96]. Furthermore, two distinct Olea europaea extracts have been shown to reduce deleterious effects induced by lead exposure in a model of neurotoxicity, involving the modulation of endoplasmic reticulum stress and the preservation of cellular redox homeostasis, pathways that are also central to the regulation of ferroptosis [96]. The potential of BPF and its synergistic combinations to modulate adipocyte ferroptosis specifically warrants further investigation, particularly given the established safety profile and metabolic benefits of these natural products in human studies.

### 6.3. Berberine, Sulforaphane, Oleuropein, and Astaxanthin: Nrf2-Activating and Iron-Modulating Agents

Beyond polyphenols, other classes of nutraceuticals offer complementary mechanisms for targeting the ferroptotic pathway. Berberine, an isoquinoline alkaloid isolated from plants such as *Berberis vulgaris* and *Coptis chinensis*, has a long history of use in traditional medicine and is recognized for its beneficial effects on glucose and lipid metabolism [83,97]. Berberine activates AMPK, modulates the gut microbiome, and has been shown to possess antioxidant and anti-inflammatory properties. Emerging evidence suggests that berberine can also modulate iron homeostasis and protect against ferroptosis by upregulating GPX4 and reducing iron accumulation, although its direct effects on adipocyte ferroptosis in obesity models remain to be fully elucidated [98,99]. A comprehensive review of natural products targeting ferroptosis in type 2 diabetes mellitus and its complications has identified berberine as one of the most promising compounds, with demonstrated efficacy in reducing ferroptosis markers in diabetic nephropathy, cardiomyopathy, and hepatic steatosis, providing a strong rationale for its investigation in the context of adipocyte ferroptosis [99]. Sulforaphane, an isothiocyanate derived from cruciferous vegetables such as broccoli, is one of the most potent naturally occurring activators of Nrf2. By chemically modifying specific cysteine residues on Keap1, sulforaphane causes a potent and sustained activation of the Nrf2-ARE transcriptional program, leading to the coordinated upregulation of a vast array of cytoprotective genes, including those directly involved in ferroptosis resistance such as GPX4, SLC7A11, and ferritin [62,81,100].

The cytoprotective role of the Keap1-Nrf2 pathway and its pharmacological activation by sulforaphane are now recognized as among the most important endogenous defense mechanisms against oxidative stress and electrophilic damage, with direct relevance to the prevention of ferroptosis in metabolic tissues [60,62]. The extraordinary potency of sulforaphane as an Nrf2 activator makes it a particularly compelling candidate for protecting adipocytes from ferroptosis under chronic oxidative stress. Oleuropein, the principal phenolic compound in extra virgin olive oil and olive leaves, is another potent Nrf2 activator with a pleiotropic profile of health benefits, including neuroprotective and cardioprotective effects [82,101]. Oleuropein has been shown to protect cells from oxidative damage, mitigate inflammation, and preserve mitochondrial function [86]. The bioactivity of olive oil phenols in neuroprotection has been specifically reviewed, highlighting their ability to activate Nrf2, inhibit NF-κB, and modulate autophagy, all of which are pathways closely tied to the regulation of ferroptosis and cellular stress responses [82]. Given that the adoption of a Mediterranean diet rich in olive oil polyphenols is associated with improved metabolic health and reduced obesity-associated inflammation, oleuropein and its metabolite hydroxytyrosol represent promising candidates for dietary ferroptosis modulation in adipose tissue [102]. The effects of polyphenol supplementation on adipose tissue morphology and gene expression in overweight and obese humans have been documented, with polyphenol-rich interventions showing the capacity to reduce adipocyte size, decrease inflammatory gene expression, and improve markers of adipose tissue function, providing clinical evidence that dietary polyphenols can directly impact the adipose organ in a manner consistent with ferroptosis protection [102]. The carotenoid astaxanthin, a red pigment found in microalgae and seafood such as salmon and shrimp, is one of the most potent natural lipophilic antioxidants known [78]. Due to its unique molecular structure, astaxanthin spans the lipid bilayer of biological membranes, positioning itself at the precise site where the lipid peroxidation chain reaction propagates during ferroptosis. Astaxanthin is a powerful direct quencher of singlet oxygen and peroxyl radicals, and it has been shown to protect against oxidative stress-induced cell death in multiple models, effects that are highly consistent with the inhibition of ferroptotic lipid peroxidation [28,78]. A mechanistic review of astaxanthin has detailed its pleiotropic biological activities, including its capacity to modulate Nrf2, inhibit NF-κB, protect mitochondrial integrity, and directly intercept lipid peroxidation chain reactions, making it one of the most comprehensively studied natural anti-ferroptotic agents [78]. Growing interest in targeting ferroptosis with natural products for treating diabetes and its complications has further validated the therapeutic potential of this approach, with multiple natural compounds demonstrating the ability to modulate GPX4, SLC7A11, Nrf2, and iron metabolism in diabetic models of tissue injury [98,99]. The nutraceuticals discussed in this section, along with their chemical classes, natural sources, and proposed anti-ferroptotic mechanisms, are cataloged in Table 3.

### 6.4. Translational Rationale and Synergistic Considerations

The points of intervention by individual nutraceuticals across the ferroptotic cascade are schematically represented in Figure 2 and summarized in Table 4.

The therapeutic rationale for deploying nutraceuticals to modulate adipocyte ferroptosis in obesity is strengthened by the concept that simultaneously targeting multiple nodes of the ferroptotic cascade may be more effective than a highly selective single-target pharmacological approach. The molecular pathogenesis of ferroptosis in the obese adipose tissue involves a network of interacting dysfunctions: iron overload, enzymatic and non-enzymatic lipid peroxidation, and a compromised GPX4-GSH defense system. A single nutraceutical, such as curcumin or quercetin, can simultaneously chelate iron, scavenge lipid radicals, and activate Nrf2, thereby addressing multiple pathogenic mechanisms in parallel. The regulation of ferroptosis by bioactive phytochemicals has important implications for medical nutritional therapy, as it provides a mechanistic foundation for the development of evidence-based dietary interventions that can modulate this death pathway in the context of chronic metabolic diseases [28]. Furthermore, the strategic combination of nutraceuticals with complementary mechanisms, for example, combining an Nrf2-activating compound like sulforaphane with a direct lipophilic radical-trapping antioxidant like astaxanthin, alongside an iron-chelating and anti-inflammatory polyphenol combination such as BPF with *Cynara cardunculus*, could achieve a synergistic blockade of the ferroptotic cascade at the levels of catalysis, propagation, and cellular defense [28,94].

The potential of nutraceutical supplementation to modulate both white and brown adipose tissue in obesity-associated disorders, through the regulation of inflammatory signaling and the promotion of thermogenic gene programs, further supports the concept that natural compounds can exert pleiotropic beneficial effects on the adipose organ that extend beyond ferroptosis inhibition to encompass the restoration of healthy adipose tissue function [88]. This nutraceutical approach, grounded in the molecular biology of ferroptosis, carries substantial translational potential for developing safe, tolerable, and mechanism-based dietary interventions aimed at protecting the adipose tissue from the pathological cell death that drives metabolic inflammation and systemic disease in obesity.

The comprehensive body of evidence from preclinical models of NAFLD, diabetes, and metabolic syndrome provides a strong translational foundation for this strategy, as many of the nutraceuticals discussed have already demonstrated efficacy in reducing ferroptosis markers and improving metabolic outcomes in animal models of obesity-related diseases [51,74,98]. However, it must be explicitly acknowledged that no nutraceutical has been tested to date for its ability to inhibit ferroptosis in the in vivo obese adipose microenvironment. The translational framework presented herein is therefore based entirely on mechanistic plausibility, derived from iron-chelating, radical-trapping, and Nrf2-activating properties characterized in non-adipose systems, rather than on direct experimental validation in obese adipose tissue. Any clinical application of these compounds for adipocyte ferroptosis inhibition should be considered hypothetical at present and must await dedicated preclinical studies using adipocyte-specific ferroptosis readouts. Rigorous preclinical studies in relevant models of obesity, employing established ferroptosis readouts specifically in adipose tissue, and, ultimately, well-designed clinical trials in humans are necessary to validate these concepts and translate them into evidence-based nutritional strategies.

**Table 3:** Nutraceuticals targeting the ferroptosis cascade.

Nutraceutical	Chemical Class	Natural Source	Proposed Anti-Ferroptotic Mechanism(s)	References
Curcumin	Curcuminoid	*Curcuma longa* (turmeric)	Nrf2 activation, iron chelation, radical scavenging, NF-κB inhibition	[75,79,87]
Resveratrol	Stilbenoid	Grapes, red wine	Nrf2 activation, ↑ GPX4, ↑ SLC7A11, ↓ ACSL4, mitochondrial protection	[80,89]
Quercetin	Flavonol	Capers, onions, berries	Nrf2/HO-1/GPX4 activation, iron chelation, lipid radical scavenging	[76,90]
Fisetin	Flavonol	Strawberries, apples	Nrf2 activation, direct antioxidant	[91]
Luteolin	Flavone	Celery, parsley, artichoke	Anti-inflammatory, antioxidant, ↓ pro-inflammatory cytokines	[92]
Bergamot Polyphenolic Fraction (BPF)	Flavonoid mix	*Citrus bergamia*	AMPK activation, Nrf2-dependent antioxidant defense, ↓ oxidative stress	[77,93,94]
Sulforaphane	Isothiocyanate	Cruciferous vegetables (broccoli)	Potent Nrf2 activation via Keap1 modification, ↑ GPX4, ↑ SLC7A11, ↑ Ferritin	[62,81,100]
Oleuropein	Secoiridoid	Extra virgin olive oil, olive leaves	Nrf2 activation, NF-κB inhibition, mitochondrial protection, autophagy modulation	[82,86,101]
Astaxanthin	Carotenoid	Microalgae, salmon, shrimp	Membrane-spanning lipophilic radical-trapping antioxidant, Nrf2 modulation, mitochondrial protection	[28,78]
Berberine	Isoquinoline alkaloid	*Berberis vulgaris*, *Coptis chinensis*	AMPK activation, ↑ GPX4, iron homeostasis modulation	[83,97,98]

Summary of the chemical classes, natural sources, proposed mechanisms of action, and key references supporting the ferroptosis-modulating properties of each compound. ↑, increased/upregulated; ↓, decreased/downregulated.

**Table 4:** Key molecular nodes of ferroptosis and corresponding nutraceutical targeting strategies.

Ferroptosis Node	Pathogenic Alteration in Obesity	Nutraceutical Intervention Strategy	Example Compounds	References
Catalytic Iron Pool	↑ Labile Fe^2+^ from uptake, sequestration, ferritinophagy	Iron chelation, ferritin upregulation (Nrf2)	Curcumin, Quercetin, BPF	[75,76,77]
Lipid Peroxyl Radical Propagation	↑ AA/AdA-PE peroxidation by LOXs and Fenton chemistry	Direct radical trapping at membrane level	Astaxanthin, Curcumin, Resveratrol	[75,78,80]
GPX4-GSH Axis	↓ GSH, ↓ GPX4 activity	Nrf2-mediated upregulation of GCLC/GCLM, SLC7A11, GPX4	Sulforaphane, Oleuropein, Resveratrol	[62,80,82]
Nrf2-Keap1 Pathway	Insufficient compensatory activation	Potent pharmacological Nrf2 activation	Sulforaphane, Curcumin, Oleuropein	[79,81,101]
Inflammatory Signaling	M1-macrophage polarization, TNF-α, NF-κB	Anti-inflammatory pathway modulation	Luteolin, Fisetin, BPF, Berberine	[91,92,93,97]
Mitochondrial Dysfunction	Altered TCA cycle, excess ROS	Mitochondrial protection, AMPK activation	Resveratrol, Oleuropein, BPF, Berberine	[83,86,89,94]

Schematic framework illustrating how specific pathogenic alterations in the ferroptotic cascade can be intercepted by nutraceutical compounds with complementary mechanisms of action, supporting the rationale for synergistic combination approaches. ↑, increased/upregulated; ↓, decreased/downregulated.

## 7. Translational Perspectives and Future Directions

The conceptual integration of ferroptosis into the molecular pathogenesis of adipose tissue dysfunction represents a significant advance with translational implications. Before discussing future directions, it is important to explicitly acknowledge the limitations of the current evidence base. As noted throughout this review, direct proof of ferroptosis in human obese adipose tissue is still lacking, and much of the mechanistic framework derives from non-adipose model systems. The gaps identified below—cell-type specificity, temporal dynamics, ferritinophagy regulation, and pathological thresholds—represent not merely academic questions but fundamental barriers to the rational development of anti-ferroptotic therapies for obesity. Addressing these gaps should be a priority for the field.

As detailed throughout this review, the unique metabolic environment of the obese adipose tissue, characterized by iron dysregulation, an abundance of peroxidation-prone PUFA-phospholipids, and a chronically besieged antioxidant defense network, creates a cellular milieu that is primed for ferroptotic death. The demonstration that signals emanating from ferroptotic adipocytes are sufficient to drive macrophage-mediated metabolic inflammation establishes a novel pathogenic axis that connects a specific form of regulated necrosis to the systemic consequences of obesity [26]. As ferroptosis research celebrates its first decade, the field has matured from the initial discovery of a novel cell death pathway to a comprehensive understanding of its emerging mechanisms, physiological functions, and therapeutic applications across a broad spectrum of diseases, providing a robust conceptual framework for its investigation in obesity and metabolic disorders [103].

This mechanistic framework opens several promising translational avenues, while simultaneously highlighting critical gaps in knowledge that must be addressed to realize the therapeutic potential of targeting ferroptosis in obesity and related disorders.

### 7.1. Ferroptosis as a Biomarker and Diagnostic Tool

The development of reliable biomarkers that reflect the extent of ferroptotic activity within adipose tissue could have significant clinical utility. Current clinical assessment of obesity-related metabolic risk relies on crude anthropometric measures such as body mass index (BMI) and waist circumference, which fail to capture the degree of adipose tissue dysfunction at the molecular level. Given the distinct biochemical signature of ferroptosis, involving specific oxidized lipid species, products of arachidonic and adrenic acid peroxidation, and alterations in systemic iron-regulatory parameters, there is potential to identify circulating markers that correlate with adipose tissue ferroptosis burden.

For example, circulating levels of lipid peroxidation products such as 4-HNE and MDA, or the ratio of oxidized to reduced forms of specific phospholipids, could potentially serve as non-invasive indicators of ongoing ferroptotic damage in the adipose organ. Similarly, the combination of elevated serum ferritin with markers of oxidative stress and inflammation might identify a specific endotype of obesity characterized by high ferroptotic activity. The cell biology of ferroptosis has advanced to the point where we now understand the subcellular localization of key ferroptosis regulators, the organelle-specific lipid metabolic pathways that control ferroptosis sensitivity, and the dynamic trafficking of iron and lipids that culminates in membrane damage, providing a detailed molecular framework for the rational design of ferroptosis-focused biomarkers [31]. The identification of such biomarkers could enable risk stratification, allowing clinicians to identify individuals who are progressing from benign to pathological obesity and who might benefit most from targeted interventions. However, significant methodological challenges must be overcome, including the development of sensitive and specific assays for ferroptosis-derived lipid mediators in biological fluids and the validation of these biomarkers against direct histological evidence of ferroptosis in human adipose tissue biopsies. The association between ferroptosis and metabolic syndrome, including its individual components such as central obesity, dyslipidemia, hypertension, and insulin resistance, has been increasingly recognized, and the development of biomarkers that can detect ferroptotic activity across these interconnected conditions could have broad clinical utility beyond obesity alone [51].

### 7.2. From Mechanism to Therapy: Pharmacological and Nutraceutical Interventions

The multi-component molecular machinery of ferroptosis presents an extensive landscape of druggable targets. The preclinical success of ferroptosis inhibitors such as ferrostatin-1 and liproxstatin-1 in models of ischemia–reperfusion injury, neurodegeneration, and other diseases has spurred interest in developing clinically viable pharmacological inhibitors of ferroptosis [31,103]. The past decade of ferroptosis research has seen an explosion in the identification of small molecule ferroptosis modulators, including not only canonical inhibitors like ferrostatin-1 and liproxstatin-1 but also compounds targeting ACSL4, system xc^−^, and Nrf2, some of which are progressing toward clinical development for specific indications [18,103]. In the context of obesity, however, chronic, long-term therapy would likely be required, placing a premium on safety and tolerability. This is where nutraceuticals, as discussed extensively in Section 6, may offer a particularly attractive bridge between mechanistic insights and clinical translation. The polypharmacological action of many natural compounds aligns well with the network-based pathogenesis of ferroptosis, where simultaneous modulation of iron homeostasis, lipid peroxidation, and Nrf2-dependent defenses may be therapeutically more robust than targeting a single node [27,28,80]. Future research should move beyond simply cataloging the antioxidant properties of nutraceuticals and toward rigorous, mechanism-driven studies that directly test their ability to inhibit adipocyte ferroptosis in relevant in vitro and in vivo models of obesity. This includes the use of established ferroptosis readouts, such as lipid peroxidation sensors, GPX4 activity assays, and iron quantification, in mature adipocytes and adipose tissue explants exposed to obesogenic stressors. The potential synergistic effects of rationally designed nutraceutical combinations, such as the pairing of an Nrf2 activator (e.g., sulforaphane or BPF) with a direct lipophilic radical-trapping antioxidant (e.g., astaxanthin) and an iron chelator (e.g., quercetin), represent a particularly promising and underexplored area that warrants systematic investigation [94]. Targeting ferroptosis with natural products is emerging as a promising therapeutic strategy for diabetes and its complications, and the lessons learned from these studies can be directly applied to the investigation of adipocyte ferroptosis in obesity, given the shared pathogenic mechanisms of iron dysregulation, oxidative stress, and metabolic inflammation [98,99].

The translational path from preclinical nutraceutical research to clinical application will require rigorous pharmacokinetic and pharmacodynamic studies, standardization of nutraceutical preparations, and the design of clinical trials with appropriate endpoints that can capture improvements in adipose tissue function and ferroptosis biomarkers.

### 7.3. Critical Knowledge Gaps: Cell-Type Specificity, Temporal Dynamics, and Molecular Regulation of Adipocyte Ferroptosis

Despite the significant advances reviewed herein, numerous fundamental questions remain regarding the role of ferroptosis in adipose tissue biology and obesity. First, the cell-type specificity of ferroptosis susceptibility within the heterogeneous adipose tissue remains entirely undefined. Are all adipocyte subtypes, white, beige, and brown, equally susceptible to ferroptosis, or do the unique mitochondrial content and lipid droplet composition of each confer differential sensitivity? White adipocytes, with their large unilocular lipid droplets and relatively low mitochondrial density, may be more vulnerable to lipid peroxidation, whereas brown and beige adipocytes, which are rich in mitochondria and uncoupling protein 1 (UCP1), might be either protected by their higher antioxidant capacity or, conversely, sensitized by their greater oxidative metabolism. No experimental data currently exist to distinguish between these possibilities. The cell biology of ferroptosis has revealed that the subcellular localization of pro- and anti-ferroptotic machinery, including the differential roles of the plasma membrane, endoplasmic reticulum, mitochondria, and lipid droplets, is a critical determinant of ferroptosis sensitivity, and these organelle-specific mechanisms remain almost entirely unexplored in adipocytes [31].

Do adipocyte progenitor cells and the stromal vascular fraction undergo ferroptosis under obesogenic conditions, and if so, what are the consequences for adipogenic capacity and tissue plasticity? Second, the temporal dynamics of ferroptosis during obesity progression remain completely unexplored. It has not been established whether adipocyte ferroptosis functions as an early trigger that initiates the inflammatory cascade in obese adipose tissue, or whether it occurs only as a late-stage consequence of severe tissue failure. This distinction has major therapeutic implications: if ferroptosis is an early event, its inhibition could prevent disease progression, whereas if it is a late feature, anti-ferroptotic interventions might offer only limited benefit once tissue dysfunction is established. Longitudinal studies in animal models—using time-resolved analyses of ferroptosis markers and inducible genetic tools to manipulate ferroptosis at different stages of diet-induced obesity—are urgently needed to address this fundamental question. Third, the molecular regulation of ferritinophagy in adipocytes is entirely unexplored. NCOA4-mediated ferritinophagy is a well-established source of labile iron that fuels ferroptosis in cancer cells and other systems [29,30,45], but whether and how this pathway operates in adipocytes, particularly under the metabolic stress of obesity, is currently unknown. It remains to be determined whether the expression and activity of NCOA4 are altered in obese adipocytes, whether ferritinophagy contributes significantly to the labile iron pool in these cells, and how HERC2-mediated regulation of NCOA4 is tuned in the context of adipose tissue iron overload. Elucidating these mechanisms could reveal adipocyte-specific therapeutic targets. Fourth, the contribution of mitochondria to adipocyte ferroptosis requires clarification. While the canonical ferroptosis pathway can proceed independently of mitochondria, recent work has implicated mitochondrial lipid metabolism, particularly the role of mitochondrial GPX4 and the electron transport chain, in modulating ferroptosis sensitivity under certain conditions [55,65]. The metabolic underpinnings of ferroptosis are increasingly understood to involve a complex interplay between cytosolic and mitochondrial metabolic pathways, with the tricarboxylic acid cycle, glutaminolysis, and mitochondrial ROS production all contributing to ferroptosis execution under specific conditions [55,65]. Given the central role of mitochondria in adipocyte thermogenesis, lipogenesis, and oxidative metabolism, understanding how mitochondrial function intersects with ferroptosis susceptibility in different adipocyte subtypes is of paramount importance. Furthermore, ferroptosis is now recognized as a nexus linking metabolism and metabolic diseases, with its core machinery—iron handling, lipid metabolism, and redox regulation—being intrinsically connected to the same metabolic pathways that are dysregulated in obesity, diabetes, and NAFLD, positioning ferroptosis as both a driver and a potential therapeutic target in these interconnected conditions [50,51]. An additional layer of complexity arises from the observation that the ferroptotic signature is reportedly diminished in the adipose tissue of obese individuals and mice, and that non-lethal ferroptotic signaling, as opposed to frank ferroptotic death, can activate a thermogenic program via a 5,15-DiHETE–Phd2–HIF1a–c-Myc–Pgc1b axis that mitigates obesity [26]. This suggests a dual, context-dependent role for ferroptosis in the adipose organ that can be reconciled by a threshold model. Transient, low-amplitude ferroptotic signals, such as the localized and controlled peroxidation of specific phospholipid species, may serve a physiological, adaptive function by activating a thermogenic program that enhances energy expenditure and mitigates weight gain, as demonstrated by Wang and colleagues. In this scenario, sub-lethal lipid peroxidation acts as a hormetic signal that triggers beneficial metabolic remodeling without causing cell death. Conversely, when pro-ferroptotic stimuli are chronic and of sufficient magnitude, as occurs in the iron-overloaded, antioxidant-depleted, and ACSL4-primed microenvironment of the pathologically obese adipose tissue, the same peroxidative cascade overwhelms cellular defense systems, resulting in lytic ferroptotic death, the release of DAMPs, and the initiation of the adipocyte–macrophage inflammatory loop described in this review. Elucidating the precise molecular thresholds that distinguish beneficial ferroptotic signaling from pathological ferroptotic execution in adipocytes will therefore be essential for designing safe and effective therapeutic strategies that inhibit pathological death without suppressing adaptive thermogenesis.

At present, these thresholds are entirely undefined. Key parameters that remain to be quantified include (i) the degree of lipid peroxidation required to initiate lytic cell death versus adaptive signaling; (ii) the intracellular labile iron concentration at which ferroptotic signaling becomes pathological; (iii) the critical loss of GPX4 or GSH activity beyond which anti-ferroptotic defenses are overwhelmed; and (iv) the role of ACSL4-mediated phospholipid remodeling in setting the threshold for lethal peroxidation. Quantitative, dose–response studies in adipocyte models are needed to establish these parameters.

This context-dependent duality should be kept in mind throughout the present review: the same molecular cascade that, at low amplitude, may support metabolic adaptation can, when chronically activated in the stressed microenvironment of the obese adipose organ, become a driver of inflammation and dysfunction. Therapeutic interventions must therefore aim to normalize, rather than indiscriminately block, ferroptotic signaling.

### 7.4. Ferroptosis in Obesity-Related Cancers and Multi-Organ Complications

The implications of ferroptosis dysregulation in obesity extend beyond metabolic disease per se. Obesity is a well-established risk factor for multiple cancer types, and the intersection between ferroptosis and cancer biology is an area of intense investigation [19,69]. The broadening horizons of ferroptosis research in cancer have revealed that ferroptosis can function as both a tumor-suppressive mechanism and a vulnerability that can be therapeutically exploited, and the chronic inflammatory, iron-rich environment of obese adipose tissue may influence cancer cell ferroptosis sensitivity in ways that impact tumor initiation and progression [69]. Adipocyte-derived signals, including those released during ferroptotic death, can influence the tumor microenvironment, potentially affecting cancer cell survival, proliferation, and metastasis. The chronic inflammatory state driven by adipocyte ferroptosis may contribute to the pro-tumorigenic milieu associated with obesity. Furthermore, the systemic iron dysregulation of obesity, with its associated elevation in serum ferritin and altered hepcidin levels, could impact ferroptosis sensitivity in distant organs, including the liver, heart, and kidney, potentially contributing to the multi-organ complications of metabolic syndrome [32,39,51]. Ferroptosis has been mechanistically linked to the pathogenesis of adiposity-based chronic disease (ABCD), a term that encompasses the full spectrum of obesity-related complications, including type 2 diabetes, NAFLD, cardiovascular disease, and certain cancers, highlighting the potential for ferroptosis-targeted interventions to address multiple obesity-related conditions simultaneously [23]. The hepatokine-adipokine-immune axis, in which the liver-derived hormone hepcidin and adipocyte-derived signals such as leptin converge to regulate systemic iron homeostasis and inflammation, represents a particularly important area for future research [36,73]. As a potential therapeutic target for obesity-related metabolic diseases, ferroptosis sits at a unique intersection of pathways that are dysregulated across multiple tissues, and interventions that can restore ferroptosis homeostasis in the adipose tissue may have beneficial ripple effects throughout the body, improving outcomes in the liver, cardiovascular system, and beyond [54]. Investigating the role of ferroptosis in the multi-organ cross-talk that characterizes advanced obesity could reveal novel pathogenic mechanisms and therapeutic targets for the prevention and treatment of obesity-related cancers and end-organ damage. The narrative of ferroptosis in obesity is still being written, and emerging evidence continues to expand our understanding of how this form of cell death contributes to adipose tissue dysfunction, metabolic inflammation, and systemic disease, promising to reveal new therapeutic vulnerabilities in the coming years [22,50].

### 7.5. Ferroptosis and the Adipocyte–Neuroimmune Axis in Obesity

An emerging frontier in ferroptosis research with particular relevance to obesity is the crosstalk between ferroptotic adipocytes and the neuroimmune system. Recent work has demonstrated that ferroptosis in the adipose tissue can engage systemic inflammatory pathways that extend beyond the local microenvironment to influence neuroimmune activation and central nervous system inflammation [72]. It has been hypothesized that this adipocyte–brain inflammatory axis could be mediated by ferroptosis-derived DAMPs and oxidized lipid mediators released into circulation from dysfunctional adipose tissue, which might cross the blood–brain barrier or act on circumventricular organs to trigger neuroinflammatory responses. The activation of microglia and the recruitment of peripheral immune cells to the hypothalamus, a key region for the regulation of energy balance, could further dysregulate appetite control, energy expenditure, and glucose homeostasis, creating a self-perpetuating loop that exacerbates obesity. This neuroimmune dimension of adipocyte ferroptosis adds a new layer of complexity to our understanding of obesity pathogenesis and suggests that the beneficial effects of anti-ferroptotic interventions may extend beyond metabolic improvements to encompass neuroprotection and the preservation of cognitive function. The intersection between adipocyte ferroptosis, neuroinflammation, and neurodegeneration represents a critical area for future investigation, particularly given the established links between obesity and increased risk of cognitive decline and neurodegenerative diseases. The concept that ferroptosis could increase obesity through crosstalk between adipocytes and the neuroimmune system provides a novel mechanistic framework for understanding the bidirectional relationship between adipose tissue dysfunction and brain health, and opens new therapeutic avenues targeting ferroptosis to simultaneously address the metabolic and neurological complications of obesity [72].

In summary, the emerging recognition of adipocyte ferroptosis as a potential contributor to adipose tissue dysfunction and metabolic inflammation has opened a fertile new frontier in obesity research. The coming years will undoubtedly witness a rapid expansion of knowledge regarding the molecular regulation of ferroptosis in the heterogeneous cellular landscape of the adipose organ. Translating these mechanistic insights into clinically viable diagnostic and therapeutic strategies, whether through targeted pharmacological agents, rationally designed nutraceutical combinations, or personalized lifestyle interventions, offers considerable potential for addressing the global burden of obesity and its devastating complications.

## 8. Conclusions

Obesity is a chronic, progressive disease driven by the pathological dysfunction of the adipose organ, and the molecular mechanisms governing adipocyte fate are central to this process. When the adaptive limits of healthy adipose tissue remodeling are surpassed, adipocyte death becomes a primary pathogenic event that triggers macrophage-driven metabolic inflammation, establishing a self-perpetuating loop that drives systemic insulin resistance and multi-organ complications.

This review has synthesized the evidence supporting the emerging role of ferroptosis, an iron-dependent, lipid peroxidation-driven form of regulated necrosis, as a potentially important mechanism of adipocyte death in obesity. The simultaneous presence of iron overload, ACSL4-esterified peroxidation-prone phospholipids, and a compromised GPX4-glutathione antioxidant axis within the obese adipose tissue creates a cellular milieu uniquely favorable to ferroptotic execution. Available evidence suggests that signals released from ferroptotic adipocytes are likely to promote macrophage polarization toward a pro-inflammatory phenotype, establishing a self-amplifying pathogenic loop that propagates local and systemic metabolic dysregulation. This adipocyte–macrophage cross-talk, amplified by the iron-sequestering phenotype of M1-polarized macrophages, may represent a novel paradigm for understanding how cell death propagates tissue dysfunction in obesity, with consequences that could extend to the neuroimmune system and multiple organ systems.

The multi-component molecular architecture of ferroptosis presents a compelling landscape for therapeutic intervention. Plant-derived nutraceuticals, including curcumin, resveratrol, quercetin, bergamot polyphenolic fraction, sulforaphane, oleuropein, and astaxanthin, possess iron-chelating, radical-trapping, and Nrf2-activating properties that can intercept the ferroptotic cascade at multiple levels. Rationally designed combinations of these compounds with complementary mechanisms represent a promising strategy for achieving synergistic protection against adipocyte ferroptosis.

While the field of adipocyte ferroptosis in obesity remains in its infancy, its integration into the molecular framework of adipose tissue dysfunction reframes our understanding of how adipocyte death drives metabolic disease. The therapeutic modulation of the adipocyte ferroptosis–macrophage inflammatory axis, particularly through mechanism-based nutraceutical interventions, has substantial translational potential to break the self-amplifying loop of cell death and inflammation that underlies the metabolic complications of obesity.

## Figures and Tables

**Table 1:** Cell death modalities implicated in obese adipose tissue.

Cell Death Mode	Key Molecular Features	Immunogenic Profile	Histological Signature in Adipose Tissue	References
Apoptosis	Caspase-dependent, cell shrinkage, apoptotic bodies	Immunologically silent (unless secondary necrosis)	Scattered apoptotic adipocytes	[1,14]
Primary Necrosis	Passive, ATP depletion, membrane rupture	Highly immunogenic (DAMP release)	Crown-like structures (CLS)	[14,15]
Pyroptosis	Caspase-1/GSDMD, NLRP3 inflammasome, IL-1β/IL-18 release	Highly immunogenic (cytokine release)	Pyroptotic adipocytes (emerging evidence)	[12,13]
Autophagy-Dependent Death	Excessive autophagy, autosis	Moderately immunogenic	Autophagic adipocytes	[30]
Ferroptosis	Iron-dependent, GPX4 inhibition, lipid peroxidation, mitochondrial shrinkage	Immunogenic (oxidized lipid mediator release)	Ferroptotic adipocytes (emerging), CLS-associated	[17,18,26]

**Table 2:** Molecular hallmarks of ferroptosis in the obese adipose tissue.

Pathway Component	Pro-Ferroptotic Alteration in Obesity	Consequence for Adipocyte Ferroptosis	References
Iron Metabolism	↑ TfR1/DMT1, ↓ Ferroportin, M1-macrophage iron sequestration, ↑ Ferritinophagy (NCOA4)	↑ Labile iron pool → Fenton reaction → •OH generation	[32,33,66]
Lipid Peroxidation Substrates	↑ ACSL4/LPCAT3 activity, ↑ AA/AdA-PE in membranes	↑ Peroxidation-prone phospholipids	[24,25]
GPX4-GSH Axis	↓ GSH (oxidative depletion), ↓ SLC7A11 (hypoxia), ↓ GPX4 activity	↓ Lipid hydroperoxide detoxification	[20,52,54]
Nrf2-Keap1 Pathway	Compensatory activation insufficient to counterbalance pro-ferroptotic stimuli	Inadequate upregulation of antioxidant/iron-regulatory genes	[63,64]
FSP1/CoQ_10_ System	Unexplored in obese adipocytes	Potential parallel defense failure	[56,57]
Mitochondria	Altered TCA cycle, ↑ glutaminolysis, ↑ mitochondrial ROS	Modulatory role in ferroptosis execution	[46,55]

Molecular hallmarks of ferroptosis in the obese adipose tissue. ↑, increased/upregulated; ↓, decreased/downregulated.

## Data Availability

No new data were created or analyzed in this study. Data sharing is not applicable to this article.

## Abbreviations

The following abbreviations are used in this manuscript:

4-HNE	4-Hydroxynonenal
AA	Arachidonic acid
ABCD	Adiposity-based chronic disease
ACSL4	Acyl–CoA synthetase long-chain family member 4
AdA	Adrenic acid
AMPK	AMP-activated protein kinase
ARE	Antioxidant response element
ATMs	Adipose tissue macrophages
ATP	Adenosine triphosphate
BAT	Brown adipose tissue
BH4	Tetrahydrobiopterin
BMI	Body mass index
BPF	Bergamot polyphenolic fraction
CLS	Crown-like structures
CoQ_10_	Coenzyme Q_10_
CoQ_10_H_2_	Ubiquinol (reduced coenzyme Q_10_)
DAMPs	Damage-associated molecular patterns
DMT1	Divalent metal transporter 1
ER	Endoplasmic reticulum
ETC	Electron transport chain
FSP1	Ferroptosis suppressor protein 1
FTH1	Ferritin heavy chain 1
FTL	Ferritin light chain
GCH1	GTP cyclohydrolase 1
GCLC	Glutamate–cysteine ligase catalytic subunit
GCLM	Glutamate–cysteine ligase modulatory subunit
GPX4	Glutathione peroxidase 4
GSH	Glutathione (reduced)
GSSG	Glutathione disulfide (oxidized)
HIF-1α	Hypoxia-inducible factor 1 alpha
IL-1β	Interleukin 1 beta
IL-6	Interleukin 6
IL-10	Interleukin 10
Keap1	Kelch-like ECH-associated protein 1
LOOH	Lipid hydroperoxides
LOX	Lipoxygenase
LPCAT3	Lysophosphatidylcholine acyltransferase 3
MDA	Malondialdehyde
M1-like	Classically activated macrophage phenotype
M2-like	Alternatively activated macrophage phenotype
NAFLD	Non-alcoholic fatty liver disease
NCOA4	Nuclear receptor coactivator 4
NF-κB	Nuclear factor kappa-light-chain-enhancer of activated B cells
Nrf2	Nuclear factor erythroid 2-related factor 2
PE	Phosphatidylethanolamine
PUFA	Polyunsaturated fatty acid
PUFA-PL	Polyunsaturated fatty acid-containing phospholipid
ROS	Reactive oxygen species
SLC7A11	Solute carrier family 7 member 11 (xCT)
TCA cycle	Tricarboxylic acid cycle
TfR1	Transferrin receptor 1
TNF-α	Tumor necrosis factor alpha
UCP1	Uncoupling protein 1
WAT	White adipose tissue
